# Cross-sensitivity analysis of land use transition and ecological service values in rare earth mining areas in southern China

**DOI:** 10.1038/s41598-023-49015-2

**Published:** 2023-12-20

**Authors:** Chenhui Zhu, Yonglin Chen, Zhiwei Wan, Zebin Chen, Jianping Lin, Peiru Chen, Weiwei Sun, Hao Yuan, Yunping Zhang

**Affiliations:** School of Geography and Environmental Engineering, Gannan Normal University, Ganzhou, 341000 China

**Keywords:** Environmental sciences, Environmental impact

## Abstract

Exploring the cross-sensitivity between land use transformation and ecological service values in rare earth mining areas is of great significance for the development of ecological protection and restoration in rare earth mining areas. To study the impact of land use changes on ecosystem service functions in rare earth mining areas, firstly, the land use change trends in the study area from 2009 to 2019 were analyzed using the land transfer matrix; then the distribution of ecosystem service values and the flow direction of ecosystem service values in the study area were measured based on the ecosystem service value equivalents; a spatial autocorrelation analysis was done on the ecosystem service values to explore their spatial distribution patterns; and finally, the cross-sensitivity coefficient was used to quantitatively assess the extent and direction of the impact of land use change on ecosystem service values. The results show that the land use types in the study area are mainly forest land and farmland, with woodland accounting for the highest proportion of the study area. The ESV changes in the study area are consistent with the trend of land use transformation, with the overall increase and decrease being comparable, and the decrease in ESV is mainly concentrated in the areas with a large increase in mining land and construction land; during the study period, the study area was significantly reduced with low—low cluster areas and the ecological environment was improved; from 2009 to 2014, the ecological sensitivity coefficient is more variable, and is more sensitive to the net conversion between water and desert, from 2014 to 2019, the ecological sensitivity coefficient is less variable, and the most sensitive is the net conversion between cultivated land and water. The study area should be reasonably developed for rare earth resources and the ecological environment around the mining area should be reasonably protected to build an ecological security pattern.

## Introduction

Land use transition refers to the dynamic evolution of surface land use elements and structures in a time series that accompanies economic and social development^1^. First proposed by the British scholar Grainger in his study of forestry-based national land use^2^, the concept of land use transition was introduced to China by Long Hualou and others and has since set off a wave of research on land use change in China^3,4^. At present, researchers have conducted more systematic studies mainly combining land use transformation with socio-economic development^5,6^, urban-rural integration^7^, landscape patterns and ecological effects^8^, but fewer studies have examined the cross-sensitivity of land use transformation and ecological service values^9^. The cross-sensitivity of ecosystem services demonstrates the bi-directional nature of land-use transition and further clarifies the intrinsic link between land-use transition and ecosystem service functions^10^.

Ecosystem services are life-supporting products and services obtained directly or indirectly through the structure, processes and functions of ecosystems, and their valuation is an important basis and foundation for ecological environmental protection^10^, ecological functional zoning^11^, environmental economic accounting and ecological compensation decisions^12^. Scholars around the world have carried out a great deal of research on ecosystem service valuation methods^13^. At present, accounting for the ecological service values (ESV) can be broadly divided into two categories, namely methods based on the price per unit of service function and methods based on the value equivalent factor per unit area^14^. The different assessment methods adopted can also lead to a wide variation in the results of the study^15^. The unit service function price approach obtains the total value from the amount of ecosystem service function and the unit price of the function volume. Such approaches model the ecosystem service function of a small area by establishing a production equation between a single service function and local ecological variables^16^. The different evaluation methods and parameters required for each service value can also affect the results of the analysis, plus the method requires many parameters and the calculation process is complex, making it difficult to calculate the results^17^. In contrast, the value equivalent per unit area factor-based approach is based on differentiating between different types of ecosystem service functions, which can be quantified in terms of the value equivalence of various service functions of different types of ecosystems, and then combined with the distribution area of the ecosystem for assessment^18^.Compared to the former, the value per unit area equivalent factor approach is more intuitive to use, requires less data and is particularly suitable for the valuation of ecosystem services in small areas^19,20^.

At the level of ESV, scholars have focused on grand scales such as global^21^, national^22^, watershed^23^, provincial^24^ and municipal scales to assess the service value of ecosystems such as forests, wetlands, watersheds, and cities. However, the study of ESV in mining areas in small areas has received less attention, especially the assessment of the service value of rare earth mining areas is scarce. By analyzing the change process of ESV in rare earth mining areas, we can explore its characteristics and problems and provide a strong basis for reclamation and ecological restoration of mining areas^25^. Fewer previous studies have combined land-use transition and the ESV, mostly leaving land-use transition to talk about the ESV, or leaving ecosystem services to talk about land-use transition. Ganzhou, Jiangxi Province is the source of ionic rare earths in China, and possesses the largest share and the most complete set of ionic rare earth resources, especially precious terbium, dysprosium, europium, yttrium, and other noble medium and heavy rare earth elements, which are honored as the "Kingdom of Rare Earths". Anyuan County and Xinfeng County are the main rare earth producing areas in Ganzhou due to their favorable mining conditions^26^. The lack of previous regulation has led to very serious uncontrolled exploitation of mineral resources. While bringing benefits to the local economy, uncontrolled exploitation has also caused a great deal of damage to the local ecosystem, resulting in various social and environmental protection problems. As environmental problems and social conflicts increase, the relevant authorities have introduced policies to rehabilitate rare earth mining areas and restrict rare earth mining^27^. During the decade 2009–2019, the Chinese Government successively launched the rehabilitation of mining areas, and promulgated the total mining volume control index for rare-earth mines, which comprehensively banned the private exploitation of mining resources. In 2015, Ganzhou Rare Earth Group shut down some of its rare earth mining sites and vetoed its proposal to go public after failing to pass an environmental assessment organized by the Ministry of Environmental Protection^28,29^. Based on this, this study takes the rare earth mining areas in Anyuan and Xinfeng counties as the study area. Based on the land use data of 2009, 2014 and 2019, the study analyses the transformation between land types in the study area, grasps the characteristics and flow direction of spatial and temporal changes of ESV in the study area, and calculates the cross-sensitivity between land use transformation and ESV, with a view to providing a scientific basis and realistic reference for optimizing the spatial pattern of land in the study area and protecting the ecological environment.

## Study area and methods

### Study area

**Figure 1:**
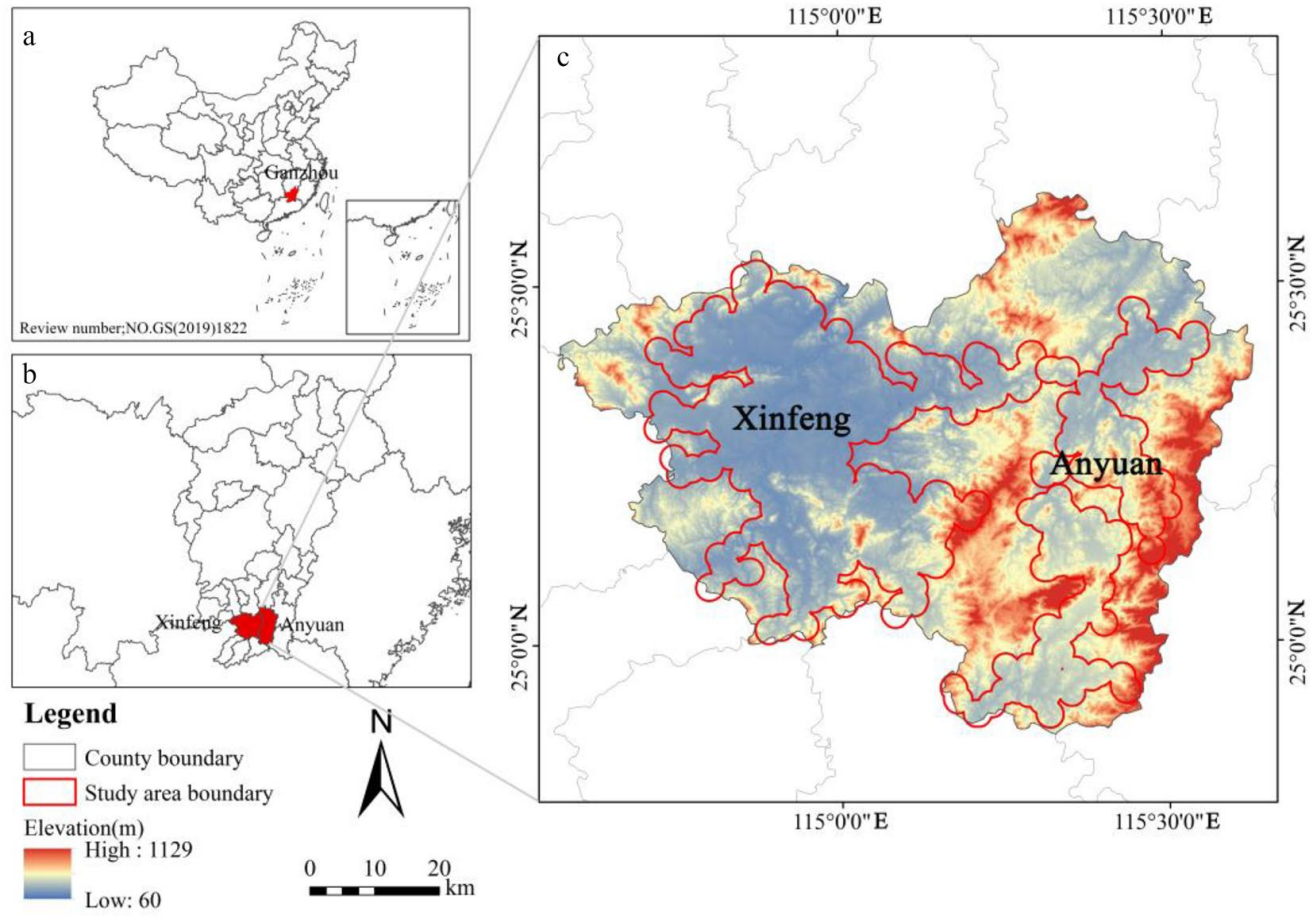
Location map of the study area (Note: the review number of the Chinese map used this time is GS (2019)1822, and the base map has not been modified.).

As one of the major production areas of rare earths, Gannan ranks among the top in the world in terms of production and reserves. Among them, the rare earth mining area is planned to cover about 2434 km^2^, and its main concentration is in the areas of Dingnan County, Longnan City, Xinfeng County, Xunwu County, Quannan County, Anyuan County, Ningdu County and Gan County. Among them, Xunwu County is dominated by light rare earths, Longnan City by heavy rare earths, and Anyuan County and Xinfeng County by medium yttrium europium-rich rare earths^30^. Two areas such as Anyuan County and Xinfeng County, the main rare earth producing areas in southern Ganzhou, were selected as the study area as shown in Fig. 1 for this study. The study area belongs to the humid monsoon climate zone on the southern edge of the central subtropics, with sufficient sunshine, high precipitation, and high forest coverage. Xinfeng County is rich in mineral resources and has a wide variety and is also one of the major resource counties in southern Ganzhou. The research has proven reserves of 420,000 tons of rare earths, with a mining area of 65 km^2^. Anyuan County has large reserves of rare earth resources, of which the heavy rare earth mining area covers 305.02 km^2^, with excellent resource conditions and a basis for development and utilization. Previous studies have been carried out only on the rare earth mining area, and there have been fewer studies on the area around the mining site. In this study, a buffer zone of 2000 m was made around the mining site, which was taken as the study area for this study. (Note: the area of the buffer zone that extends beyond the blank area of the administrative boundary was not included in this study.)

### Data source

The land use data of the study area for the three periods from 2009 to 2019 were obtained from the Jiangxi Provincial Land Use Change Survey database and provided by the relevant departments. Socioeconomic data were obtained from the Anyuan and Xinfeng Statistical Yearbook (2009–2020). Digital elevation Model (DEM) was gathered from the Geospatial Data Cloud website (http://www.gscloud.cn/).

### Methods

#### Classification of land use types

**Figure 2:**
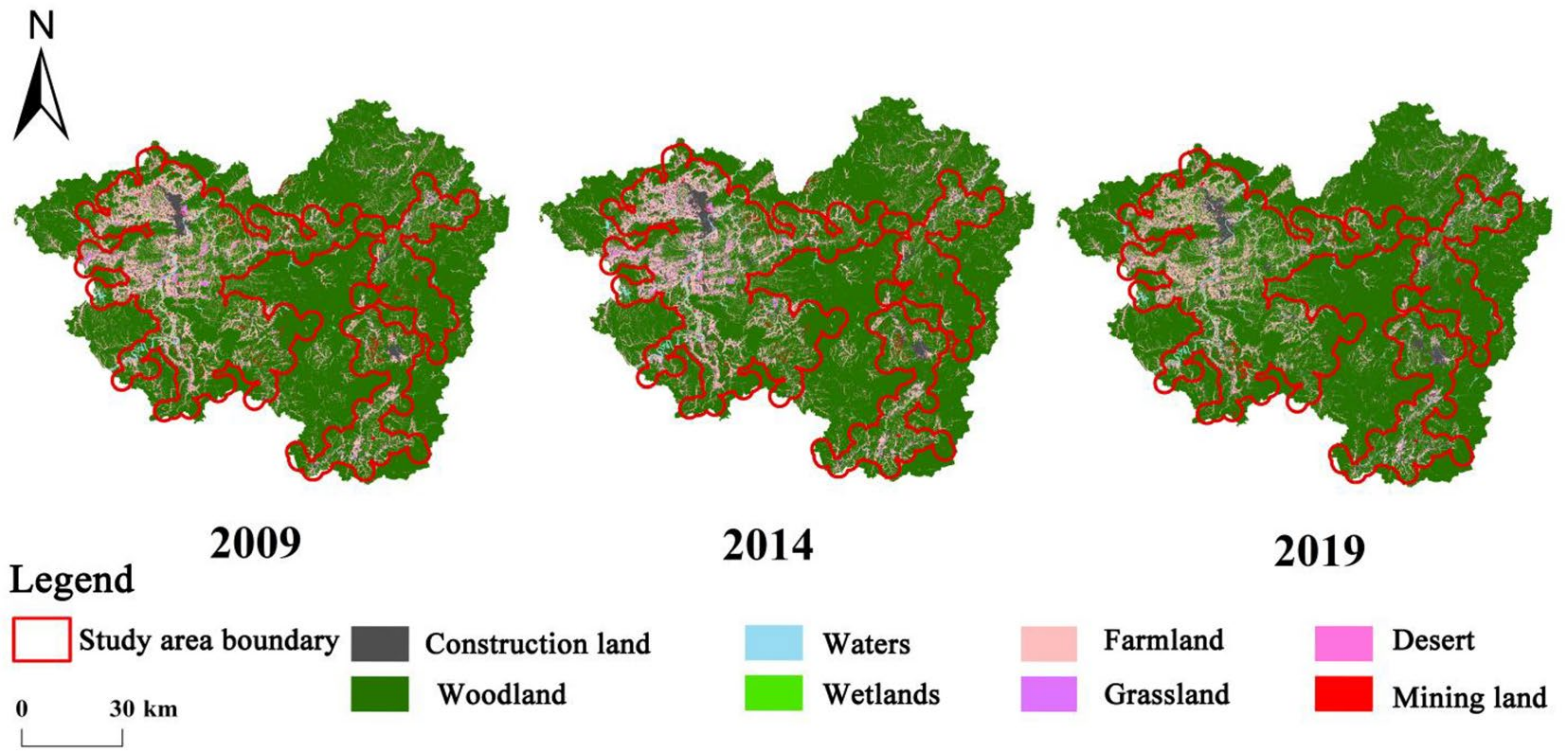
Map of current land use in the study area in 2009–2019.

Based on the classification of the land use change survey and according to the characteristics of the study area, this study further integrated the land use types in the study area into eight land types, including forest land, grassland, farmland, desert, water, wetland, construction land and mining land (Tables 1 and 2). Considering the characteristics of the land use change survey data such as the fineness and complexity of the distribution of land class patches, it is difficult to process the vector data of the study area at one time. Therefore, with the help of GIS software, the rare earth mining areas in the study area were extracted separately, named as mining sites, and processed one by one. The vector data of the study area was also transformed into raster data with a spatial resolution of 30 m to better reflect the actual situation of the study area and subsequent processing. The current status of land use in the study area from 2009 to 2019 is shown in Fig. 2.

**Table 1:** Classification of land use types based on the Second National Land Surveys.

Reclassify	Land use types
Farmland	Paddy land, dry land
Woodland	Shrublands, other woodlands, bamboo woodlands, tree woodlands, orchards, other gardens, tea gardens
Grassland	Other grassland
Construction land	Villages, landscapes and specific sites, highway sites, established towns, rural roads, facility agricultural sites, waterworks and construction sites, railway sites
Wetlands	Inland mudflats
Waters	Ditches, river surface, reservoir surface
Desert	Bare ground
Mining land	Mining land

**Table 2:** Classification of land use types based on the Third National Land Surveys.

Reclassify	Land use types
Farmland	Paddy land, dry land
Woodland	Tree woodland, bamboo woodland, other woodland, shrubland, orchards, other gardens, tea gardens
Grassland	Other grassland
Construction land	Rural residential land, special land, highway land, rural roads, agricultural land for facilities, waterworks land, railway land, urban road land, institutional land, transportation service station land, science, education, culture and health land, utility land, plaza land, industrial land, commercial service land, residential land, parks and green spaces, logistics and warehousing land, pipeline transport land
Wetlands	Inland mudflats
Waters	Ditches, river water surface, pit pond water surface, breeding pond, reservoir water surface
Desert	Bare ground
Mining land	Mining land

#### Ecosystem service values measurement models

Based on the actual reality of the study area, the value of each service equivalent in the study area was assigned based on the ESV assessment system proposed by Costanza^21^ and with reference to the Chinese terrestrial ecosystem service equivalent factor table established by Xie Gaodi et al^22,23^, with appropriate corrections (as shown in Table 3). The calculation equations were as follows:

1 $$ESV = \sum\nolimits_{i = 1}^{{\text{n}}} {A_{i} } \times VC_{i}$$

2 $$VC_{i} = \sum\nolimits_{j = 1}^{k} {EC_{j} } \times E_{a}$$

in the formula, ESV is the ecosystem service values, n is the total number of ecosystem types, *i* is the ecosystem type,* j* is the ecosystem service type, *A*_*i*_ is the area of ecosystem type *i*, *VC*_*i*_ is the ecosystem service values per unit area of ecosystem type *i*, *EC*_*j*_ is the value equivalent of the *j*th ecosystem service of a particular ecosystem type, and E_a_ is the economic value of 1 unit of ecosystem service.

**Table 3:** Table of ecosystem service equivalent per unit area of land use in the study area.

Level 1 classification	Level 2 classification	Woodland	Farmland	Wetlands	Construction land	Grassland	Desert	Waters	Mining land
	Food production	0.29	1.36	0.51	0	0.38	0	0.8	0
Supply services	Raw material production	0.66	0.09	0.5	0	0.56	0	0.23	0
	Water supply	0.34	−2.63	2.59	0	0.31	0	8.29	−5.5
	Gas regulation	2.17	1.11	1.9	−1.3	1.97	0.19	0.77	0
Regulation services	Climate regulation	6.5	0.57	3.6	−3.2	5.21	0.18	2.29	0
	Purification of the environment	1.93	0.17	3.6	0	1.72	0.21	5.55	−2.56
	Hydrological regulation	4.74	2.72	24.23	0	3.82	0.22	102.24	−3.63
	Soil conservation	2.65	0.01	2.31	−1.2	2.4	0.34	0.93	−2.9
Support services	Maintenance of nutrient cycles	0.2	0.19	0.18	0	0.18	0.11	0.07	−2.2
	Biodiversity	2.41	0.21	7.87	−3.2	2.18	0.26	2.55	−1.2
Cultural services	Aesthetic landscape	1.06	0.09	4.73	0.2	0.96	0.07	1.89	0
	Waste degradation	0.23	0.4	0.02	−1.2	0.05	0	0.08	−5.09
Ecological service	Soil erosion	0	0	0	−3.8	0	−1.3	0	−3.39
	Environmental restoration	1.95	0.3	4	−1.51	1.61	0.02	9.71	−4.85

According to the latest abandoned mine ecological restoration project (2021–2035) announced by the people's governments of Anyuan and Xinfeng counties, restoration of 1 ha costs CNY 266,400, which is used to include topographic and geomorphological remodeling works, infrastructure works, ecological restoration and protection works, reclamation works, and supporting works. The coefficients of waste degradation, soil erosion and environmental restoration of mining land in ecological services are determined according to the proportion of the spent funds in the project and the expert scoring method.

The economic value of annual food production per unit area of farmland was corrected by consulting the 2009–2020 statistical yearbook of the study area and combining the land use structure of the study area and the actual economic situation of the study area: the average food production in the study area from 2009 to 2019 was 5360.12 kg/ha, with reference to the average annual food price of yuan 5.39/kg in the study area in 2019 as the benchmark. Excluding the human input situation, the economic value of food production in the study area was obtained as 4127.32 CNY/ha according to the Chinese ecosystem service coefficient correction method proposed by Xie Gaodi, i.e. an ESV equivalent is 1/7 of the food production value per unit area, to obtain the regional ecosystem service values equivalent, and on the basis of the regional ESV equivalent table (Table 3), the study the ESV in the study area was obtained on the basis of the regional ecosystem service equivalent table.

#### Coefficient of cross-sensitivity

The Coefficient of Cross-Sensitivity (CCS) is the rate of change in ESV per unit area when a land use type is converted to another land type^27,28^. The Coefficient of Cross-Sensitivity (CCS) is used to indicate the impact of inter-conversion between different land types on the overall ESV and the land use type undergoing conversion, which is directional in nature. For example, the conversion between woodland and farmland is both a conversion of woodland to farmland and a conversion of farmland to woodland. This two-way conversion may result in different amounts of change in the ESV depending on natural endowments and geographical location. However, when the study area is small, according to the ESV estimation model proposed by Xie Gaodi et al^22^, the equivalent two-way conversion can offset each other. Therefore, the Coefficient of Cross-Sensitivity (CCS) is used to measure the cross-sensitivity of ecosystem services to land-use change with reference to related studies^31^, its equation is as follows:

3 $$CCS\left( {x,y} \right)_{ki} = \left| {\frac{{\Delta ES_{{\left( {x,y} \right)}} }}{{\Delta CCL_{{\left( {k,i} \right)}} }}} \right| = \left| {\frac{{(ES_{x} - ES_{y} )/ES_{y} }}{{(IR_{{\left( {k,i} \right)}} - TR_{{\left( {k,i} \right)}} )/(A_{k} + A_{i} )}}} \right|$$

where: *CCS*(*x, y*) is the coefficient of cross-sensitivity of the transition between land use type *k* and land use type *i* in year* x*;*△ES*_*(x, y)*_ is the rate of change in ESV from year *x* to year *y*; *△CCL*_*(k ,i)*_ is the net transition rate of land use types *k* and *i*; *IR*_*(k, i)*_ is the area of transition from land use type* k* to land use type *i*; *TR*_*(k, i)*_ is the area of transition from land use type *i* to land use type* k*; A_*k*_ and A_*i*_ are the base period areas of land use types *k* and *i* respectively. If CCS *(x, y)* > 0 indicates that the change in ESV increase or decrease is changing in the same direction as the net transition of different land use types; if CCS *(x, y)* < 0 indicates that the change in ESV increase or decrease is changing in the opposite direction as the net transition of different land use types; the larger the value of |CCS *(x, y)* |, the more sensitive the ESV is to the net transition of a certain two land use types, and the smaller the less sensitive.

#### Land use transfer matrix

Referring to the rare earth mining area extent line of Anyuan County and Xinfeng County and using the buffer zone analysis of GIS software for the mining land in the study area in 2009, the buffer zone distance was set to 2000 m as the study area extent for this study. Using the cropping tool and data extraction function of GIS software, the data from the reclassified land survey data of the study area in 2009, 2014 and 2019 were cropped using the rare earth mining area extent line to generate the current land use data of the rare earth mining area for three periods from 2009 to 2019. A comparative analysis was also conducted through the statistical table of land use types of area to explore the current land use status and its change characteristics in the rare earth mining area of the study area from 2009 to 2019.

Using the intersection tool of GIS software, the study area rare earth mining area 2009–2019 land use status map was intersected and analyzed, after which the attribute table data was imported into Excel and inserted into the pivot table, the study area land transfer matrix for 2009–2014 and 2014–2019 could be derived. The transfer matrix equation is as follows^32^:

4 $${\text{S}}_{ij} = \left( {\begin{array}{*{20}c} {S_{11} } & {S_{12} } & \ldots & {S_{1n} } \\ {S_{21} } & {S_{22} } & \ldots & {S_{2n} } \\ \ldots & \ldots & \ldots & \ldots \\ {S_{n1} } & {S_{n2} } & \ldots & {S_{nn} } \\ \end{array} } \right)(ij = 1,2,3,4, \ldots ,n)$$

where S_*ij*_ is the area of the study area where land type *i* at the beginning of the study area is transformed into land type *j* at the end, *i* and *j* are the land use types of the study area at different time periods (*i, j* = 1, 2, 3, 4, …, n), and *n* is the land use type.

#### Ecosystem service values flows

The flow of ecosystem service values can visually reflect the transformation of ecosystem services in the study area^32,33^. This study uses land type conversion data to calculate the ecosystem service value gains and losses due to the conversion of different land types to each other, with the aim of analyzing the impact of land type conversion on its service value within the rare earth mining area. The calculation equation is as follows:

5 $${\text{PL}}_{ij} = \, (VC_{j} - VC_{i} )A_{ij}$$

where: PL_*ij*_ is the ecosystem service value gain or loss after the conversion of ecosystem type *i* to ecosystem type *j*, VC_*i*_ and VC_*j*_, are the ecosystem service value coefficients for ecosystem type i and ecosystem type *j* respectively, and A_*jj*_ is the area of ecosystem type *i* converted to ecosystem type *j*.

#### Characteristics of spatial variation in ESV

By analyzing the ESV in spatial correlation analysis, we explored its distribution characteristics in space. In this study, Global Moran's I and Local Moran's l were used to test whether there is a spatial clustering effect of ESV between the study area and the surrounding areas from 2009 to 2019^34,35^. The formula is as follows:

6 $$Moran^{\prime}s \, I = \frac{{\sum\limits_{i = 1}^{n} {\sum\limits_{j = 1}^{n} {w_{ij} \left( {x_{i} - \overline{x}} \right)} } \left( {x_{j} - \overline{x}} \right)}}{{S^{2} \left( {\sum\limits_{i} {\sum\limits_{j} {w_{ij} } } } \right)}}$$

where: *n* is the number of spatial units,* x*_*i*_ and *x*_*j*_, denote the observed values of unit *i* and unit *j*, respectively, (*x*_*i*_ − *x*_*j*_) is the deviation of the observed value on the ith spatial unit from the mean, w_ij_ is the spatial weight matrix established based on the spatial *k*-neighbourhood, and S^2^ denotes the variance.

7 $$S^{2} = \frac{1}{n}\sum\limits_{i = 1}^{n} {\left( {x_{i} - \overline{x}} \right)^{2} }$$

8 $$Moran^{\prime}s \, I_{i} = \frac{{\left( {x_{i} - \overline{x}} \right)\sum\limits_{j = 1}^{n} {w_{ij} \left( {x_{i} - \overline{x}} \right)} }}{{S^{2} }}$$

where: Moran'sI represents the local spatial autocorrelation Moran's index, and the rest has the same meaning as Eq. (6).

## Results

### Analysis of land use status and transition

#### Analysis of the current land use situation

As shown in Table 4, the farmland area in the study area showed a continuous decreasing trend from 2009 to 2019, with a net decrease of 10,421.42 ha during the decade; the study area had the highest proportion of woodland, which showed a decreasing trend followed by an increasing trend, with a net increase of 3940.44 ha; during this period, the grassland and desert in the study area showed a continuous decline; the construction land showed a continuous increase, with a larger increase in the later part of the study period (2014–2019), with a net increase of 6957.05 ha; the area of wetland in the study area showed a trend of first decrease and then increase, with the largest decrease in the pre-study period (2009–2014), when the area decreased by 3858.88 ha, and a net increase of 950.42 ha during the decade; the area of waters in the study area showed an increasing trend followed by a decreasing trend, with a net increase of 2784.15 ha; the mining land in the study area also showed a continuous decreasing trend, with a net decrease of 717.95 ha over the study period.

**Table 4:** Status of land use in the study area 2009–2019 (unit: ha).

Land classes	2009		2014		2019	
	Area	Percentage (%)	Area	Percentage (%)	Area	Percentage (%)
Farmland	55,273.17	18.7	55,142.30	18.6	44,851.75	15.1
Woodland	206,494.48	69.7	204,633.90	69.1	210,434.92	71.0
Grassland	5038.91	1.7	4627.45	1.6	2455.88	0.8
Construction land	16,292.93	5.5	19,081.19	6.4	23,249.98	7.8
Wetlands	4487.07	1.5	628.19	0.2	5437.49	1.8
Waters	5077.29	1.7	8828.27	3.0	7861.44	2.7
Desert	1098.89	0.4	975.77	0.3	196.53	0.1
Mining land	2552.49	0.9	2398.14	0.8	1834.54	0.6

#### Analysis of land use transition

**Figure 3:**
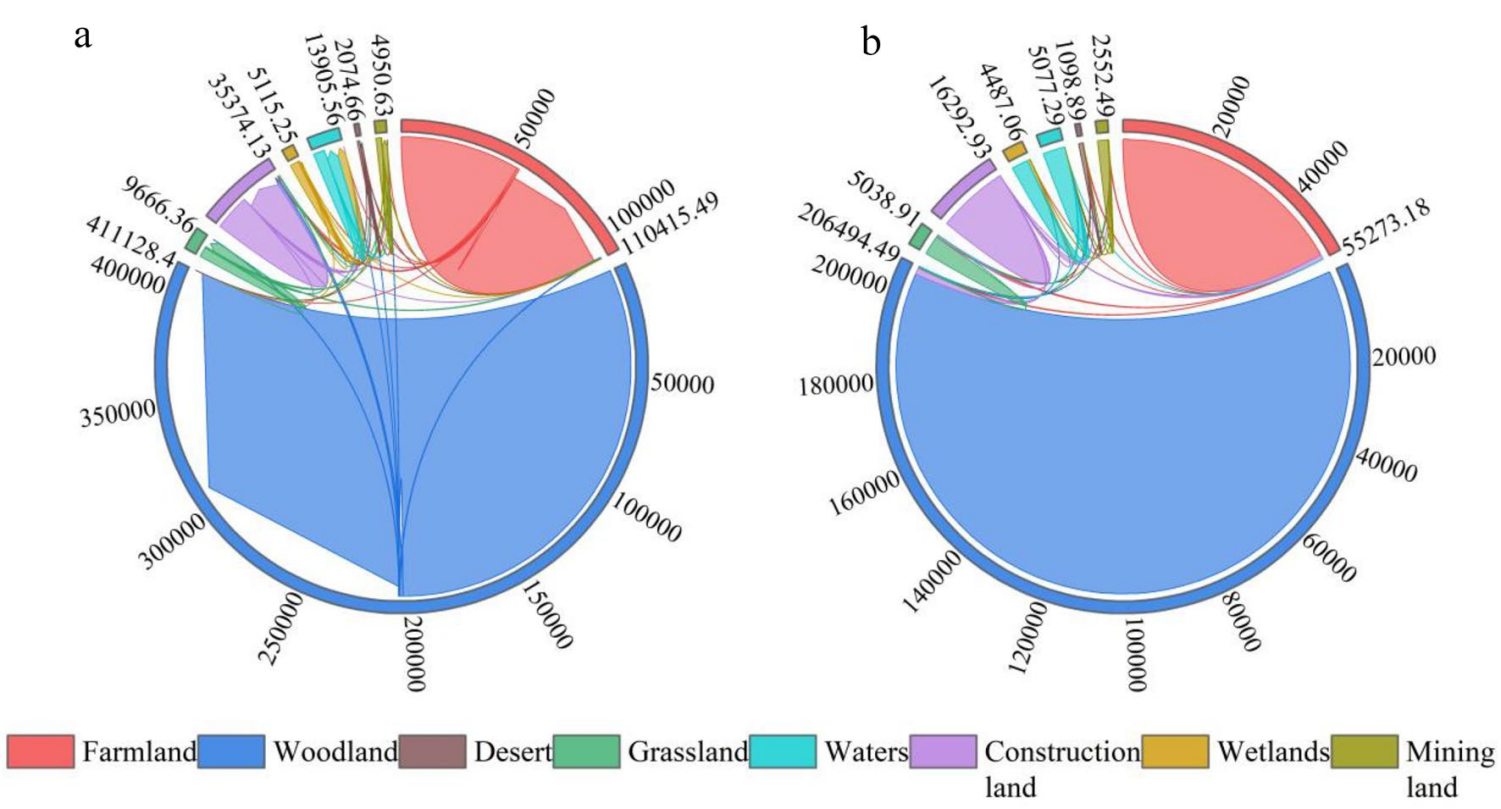
Chord diagram of the land transfer matrix in the study area 2009–2014 ((**a**) is a chord diagram, (**b**) is a scaled chord diagram).

The conversion of mining land to farmland was the largest in the study area over the 5-year period 2009–2014 (Fig. 3), with a conversion of 103.05 ha, followed by construction land and woodland, with a total reduction of 154.35 ha in mining land over the period. The conversion of farmland to construction land was the largest in the study area, followed by woodland, desert and mining land. During this period, farmland was converted to other land use types by 1123.60 ha and other land use types were converted to farmland by 992.73 ha. The largest area of woodland was converted to construction land, followed by farmland and grassland. The conversion of wetlands to water areas was large, with a total conversion of 3765.08 ha, which also led to a significant decrease in wetlands during the study period. The conversion of areas of waters to other land use types was low, which is also related to the nature of waters resources. The conversion of desert to farmland was high and the conversion to other land use types was low.

**Figure 4:**
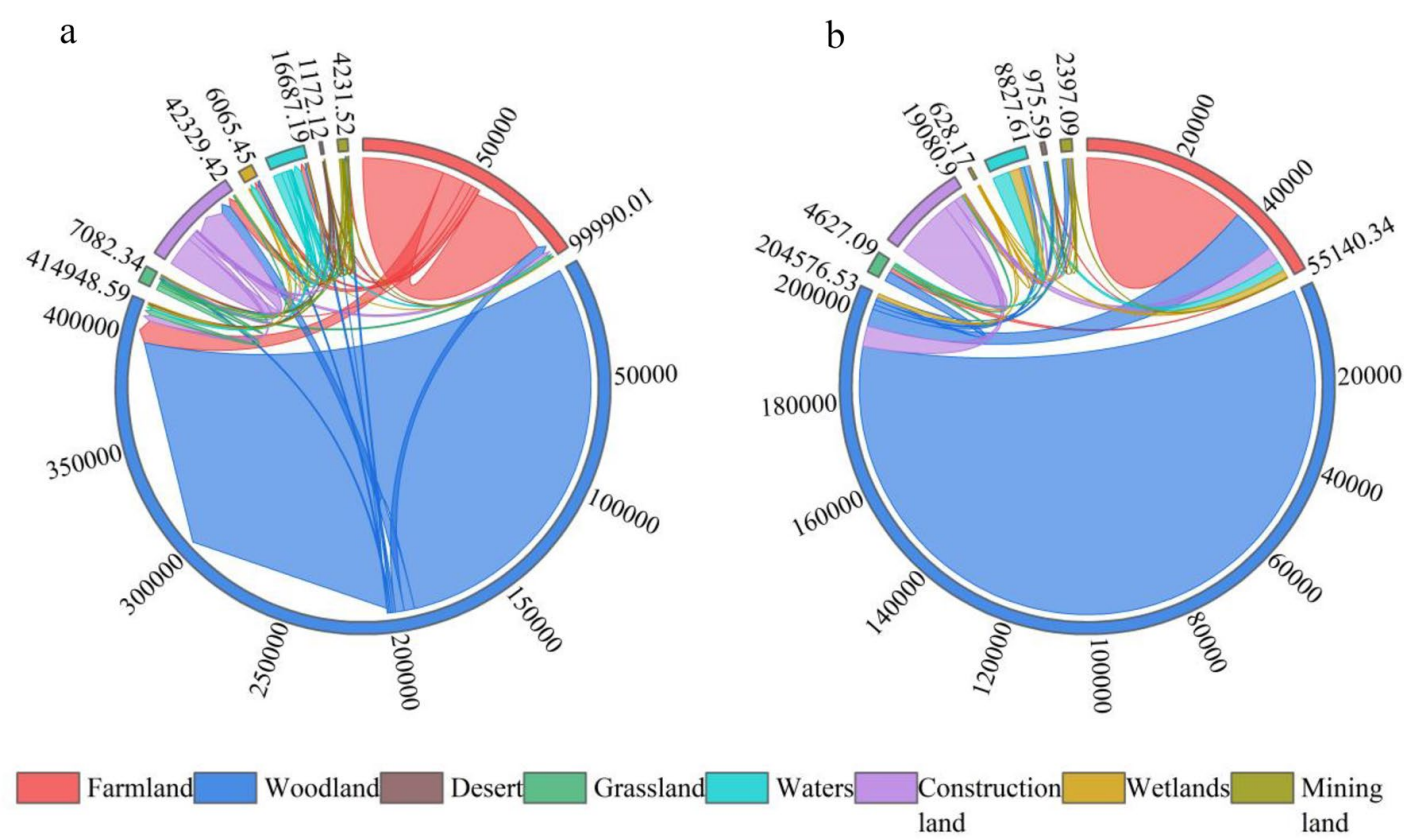
Chord diagram of the land transfer matrix for the study area 2014–2019 ((**a**) is a chord diagram, (**b**) is a scaled chord diagram).

From 2014 to 2019 (Fig. 4), the largest area of mining land was converted to woodland in the study area, converting 1024.36 ha, followed by construction land and grassland, and the largest area of woodland was converted to mining land, converting a total of 1013.57 ha. During this period, mining land was reduced by a total of 562.67 ha. The conversion of farmland to other land use types amounted to 18,206.60 ha and other land use types to farmland amounted to 7915.93 ha. The largest area of woodland was converted to construction land, followed by farmland and waters, with a total increase in woodland area of 5795.53 ha. The conversion of grassland to woodland and farmland amounted to 2240.70 ha, with a total decrease in grassland area of 2171.81 ha. The conversion of construction land to woodland and farmland was higher, at 2730.21 ha and 1834.07 ha respectively, with a total increase of 2788.26 ha in construction land. The conversion of waters to wetlands was the largest, followed by woodland, with a decrease in waters of 968.03 ha. The conversion of desert to woodland was high, with less conversion to other land types, with a decrease in desert of 562.67 ha during the study period.

### Ecological service values in 2009–2019

The ESV in the study area was calculated by the value per unit area equivalent factor method (Table 5). The service value of each type of ecosystem was divided by the sum of the ESV for the year to obtain the proportion of the service value of each type of ecosystem. Because the ESV for both mining land and construction land is negative, their share is also negative to illustrate the negative impact on the overall ESV. The greater the absolute value of the negative value, the greater the negative impact. The total ESV in the study area was CNY 25.43 billion, CNY 26.25 billion and CNY 26.85 billion in 2009, 2014 and 2019 respectively. This is mainly due to the decrease in the area of forest land and arable land and the increase in the area of construction land.

**Table 5:** ESV in the study area in 2009–2019 (unit: CNY billion).

Land classes	2009		2014		2019	
	ESV	Percentage (%)	ESV	Percentage (%)	ESV	Percentage (%)
Farmland	1.05	4.12	1.04	3.98	0.85	3.16
Woodland	21.42	84.21	21.22	80.86	21.83	81.30
Grassland	0.44	1.75	0.41	1.55	0.22	0.81
Construction land	−1.02	−4.02	−1.20	−4.56	−1.46	−5.44
Wetlands	1.04	4.08	0.15	0.55	1.26	4.68
Waters	2.84	11.16	4.93	18.80	4.39	16.36
Desert	0.00	0.01	0.00	0.00	0.00	0.00
Mining land	−0.33	−1.30	−0.31	−1.18	−0.24	−0.88

In terms of the ESV of different ecosystems, woodland and waters are the main contributors to the ESV of the entire study area. During the study period, the ESV of farmland, grassland, desert, and mining land showed a continuous decreasing trend, while those of woodland and wetlands showed a decreasing trend followed by an increasing trend, and those of waters showed an increasing trend followed by a decreasing trend.

### Spatial and temporal distribution characteristics of ESV

**Figure 5:**
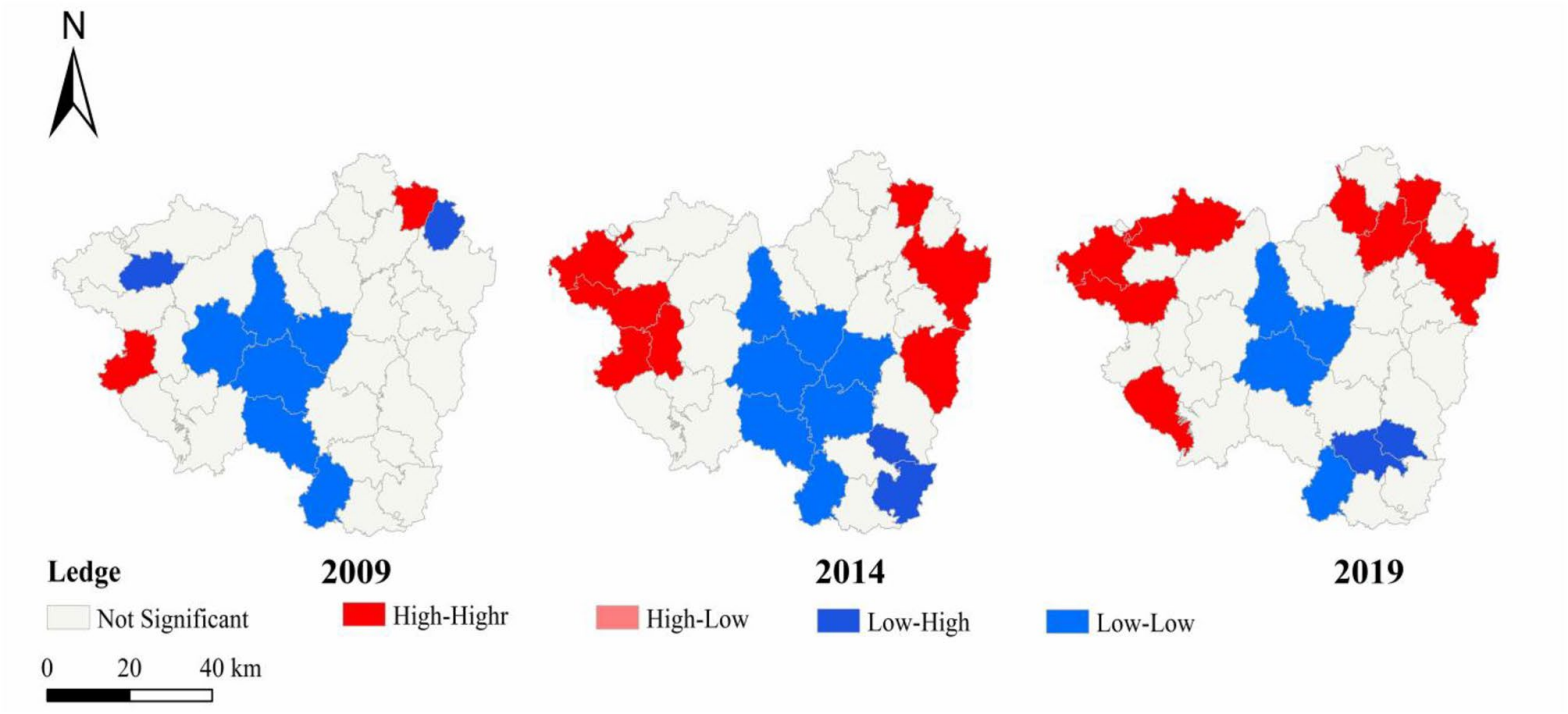
LISA clustering of ESV in the study area and surrounding towns in 2009–2019.

To investigate the spatial and temporal changes in the ESV of the rare earth mining area, the LISA (Local indicators of Spatial association) clustering map of the ESV of the study area and the surrounding towns was calculated using Geoda software^35^. As can be seen from Figure 5, during the study period, the spatial distribution of ESV in the study area and the surrounding towns varied considerably, with a higher number of High-High Cluster and Low-Low Cluster, a lower number of Low-High Outlier and High-Low Outlier, and the overall spatial distribution level of ESV in the study area and the surrounding towns was dominated by similar types of agglomerations. The spatial distribution of ESV in the study area and surrounding towns in the western and eastern regions showed a High-High Cluster, while the central region was dominated by a Low-Low Cluster, indicating that there are significant differences in the level of green ecological development between the central region and the eastern and western regions, and that there is a serious imbalance in the spatial distribution of ESV. During the study period, the overall clustering of ESV in the study area and the surrounding towns changed considerably, with fewer Low-Low Clusters in the central region and more High-High in the eastern and western regions, suggesting that during the period of 2009-2019, the clustering of ESV of the study area and the surrounding towns with High-High values was more pronounced, and that the imbalance of the distribution of clusters was improved, and the pressure of ecological level deterioration on the central region was reduced.

### Ecosystem service values flows

#### ESV gain and loss flows in 2009–2014

From 2009 to 2014 (Table 6), only the ESV of grassland increased, while the ESV of other land types decreased. The ESV of mining land shifted to construction land the most in the study area, followed by farmland. In the process of shifting ESV of mining land, woodland and grassland contributed the most, and the ESV of woodland shifted to mining land was the highest. The ESV of farmland in the study area was the most shifted to construction land, with a total of CNY 39,471,800 shifted to construction land. During this period, the ESV of other land use types shifted to farmland was CNY 15,938,600, with a total decrease of CNY 23,264,200 in farmland. Woodland had the most ESV shifted to construction land, followed by grassland and farmland. Construction land, grassland, wetlands, desert and, as with mining land, most of the ESV shifted to farmland. The shift in ESV from waters to other land types is less frequent and is closely related to the stability of the waters themselves.

**Table 6:** ESV gain and loss flows in 2009–2014 (unit: CNY million).

	2014								
	Land classes	Farmland	Woodland	Grassland	Construction land	Wetlands	Waters	Desert	Mining land
2009	Farmland	86,939	19	0	−3947	2	37	4	−36
	Woodland	458	328,429	1699	−4952	0	98	7	−351
	Grassland	704	5	35,907	−618	0	33	1	−38
	Construction land	50	12	0	−58,360	0	47	0	−3
	Wetlands	21	1	0	−283	13,486	195,196	0	−11
	Waters	0	0	0	−63	0	262,280	0	−2
	Desert	196	1	0	−65	0	0	624	−9
	Mining land	165	78	0	−228	0	0	0	−17,355

#### The ESV gain and loss flows in 2014–2019

As indicated in Table 7, the ESV of grassland and wetlands increased and those of farmland, desert, waters, mining land, woodland and construction land decreased from 2014 to 2019. The ESV of mining land shifted to wetlands the most in the study area, followed by grassland and woodland. In the transfer of ESV from mining land, woodland and farmland contributed most to the process. The ESV of cultivated land in the study area shifted to waters the most, with a total shift to waters of CNY 116,251,500. During the period, the ESV of other land use types shifted to farmland of CNY 127,092,500, with a total decrease of CNY 140,643,000 in farmland. The ESV of woodland, construction land, grassland, wetlands, and waters were all shifted to waters the most, and the ESV of waters were shifted to wetlands the most. The ESV of desert shifted most to grassland and woodland, followed by waters and wetlands.

**Table 7:** ESV gain and loss flows in 2014–2019 (unit: CNY million).

	2019								
	Land classes	Farmland	Woodland	Grassland	Construction land	Wetlands	Waters	Desert	Mining land
2014	Farmland	59,298	16,582	427	−13,456	34,886	116,251	19	−1358
	Woodland	6362	308,550	6605	−16,911	16,798	55,927	24	−7526
	Grassland	1670	3598	5473	−1188	3519	7002	9	−221
	Construction land	2945	4383	3438	−48,741	5108	10,602	10	−463
	Wetlands	59	274	131	−115	911	16,862	0	−41
	Waters	1240	1916	1089	−1315	53,224	199,270	1	−283
	Desert	227	811	887	−283	367	413	65	−127
	Mining land	207	1645	1904	−1472	1928	1141	2	−3602

### Cross-sensitivity analysis of ecosystems

**Figure 6:**
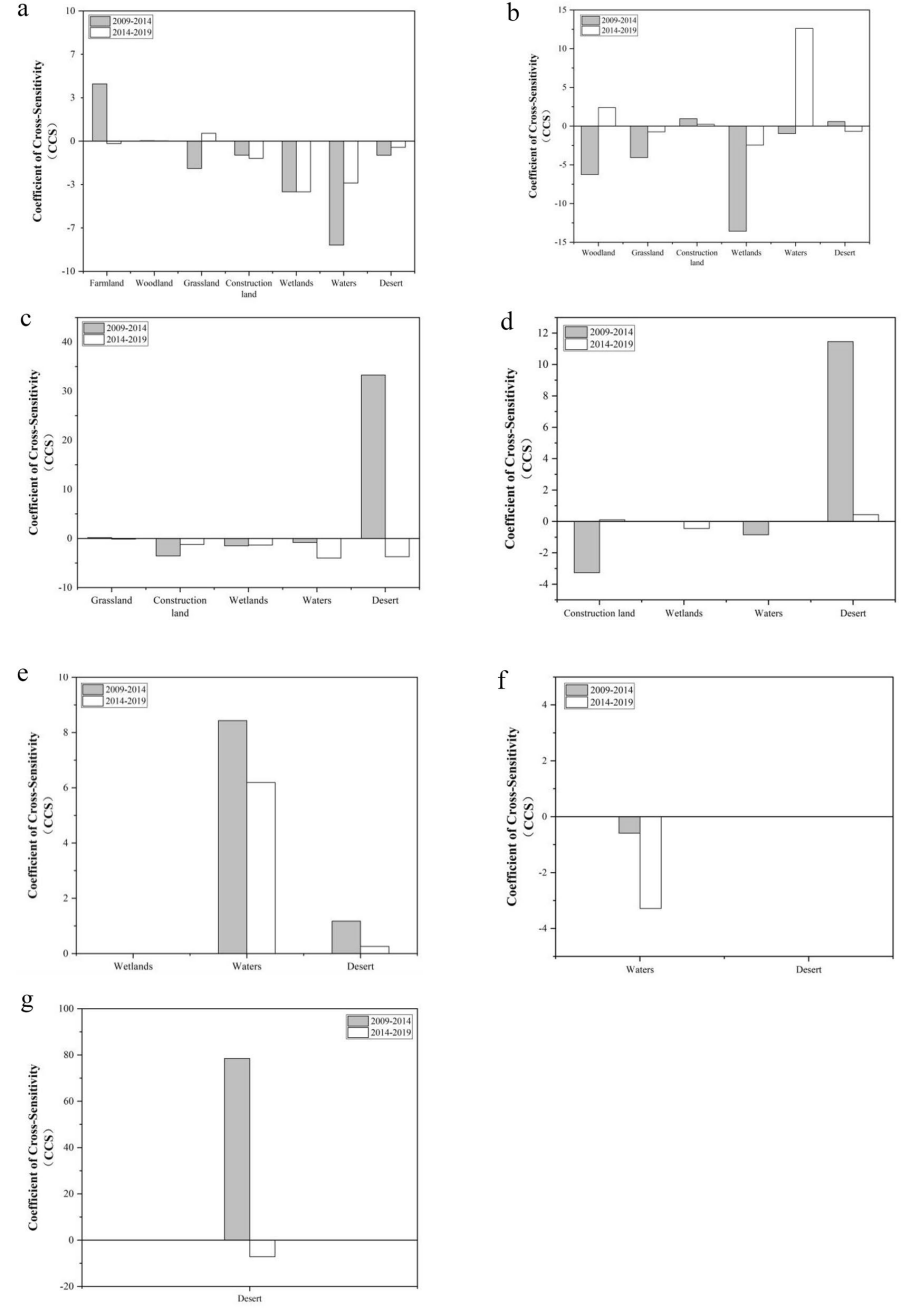
Cross-sensitivity coefficients between land-use types: (**a**) cross-sensitivity of mining land to other land class conversions; (**b**) description of what is contained in the second panel. (**c**) Cross-sensitivity of woodland to other land class conversions; (**d**) cross-sensitivity of grassland to other land class conversions; (**e**) cross-sensitivity of construction land to other land class conversions; (**f**) cross-sensitivity of wetlands to other land class conversions; (**g**) cross-sensitivity of waters to other land class conversions.

From 2009 to 2014, the ecological sensitivity coefficient varied considerably and was more sensitive to the net conversion between waters and deserts, with a Coefficient of Cross-Sensitivity (CCS) value of 78.5 for both; from 2014 to 2019, the ecological sensitivity coefficient varied less and was most sensitive to the net conversion between farmland and waters, with a CCS value of 12.62 for farmland and waters. The main results of the analysis are as follows:

The CCS of mining land was cross-sensitive to conversion to other land types (Fig. 6a). In the early part of the study (2009–2014), the CCS values for the conversion of mining land to grassland, construction land, wetlands, desert, and waters were negative, indicating that the conversion of mining land to the above-mentioned land types was more inhibitory to changes in ESV, with the absolute value of CCS for waters being large, indicating that the conversion of mining land to waters was the most inhibitory to changes in its own ESV. Later in the study (2014–2019), the CCS values for mining land with woodlands and grasslands were positive and with other land types were negative, indicating that the net change from mining land to woodland and wetlands contributed to the change in ESV. During the study period (2009–2019), the CCS values for mining land versus farmland changed from positive to negative, indicating that the conversion of mining land to woodland and wetlands contributed to the change in ESV. During the study period (2009–2019), the CCS values for mining land versus farmland changed from positive to negative, indicating that the conversion of mining land to farmland changed from a facilitative to an inhibitory effect on changes in ESV. Conversely, the CCS values of mining land and grassland changed from negative to positive, indicating that the conversion of mining land and grassland changed from an inhibitory to a facilitative effect on the change of ESV. The CCS value between mining land and waters changes from −7.98 to −3.21, indicating that the transition between mining land and waters has an inhibitory effect on the change in ESV and that the ecosystem sensitivity to such a transition gradually decreases. Similarly, the same is true for the conversion between mining land and desert. The change in CCS from −1.08 to −1.32 for mining land to construction land indicates a gradual increase in the sensitivity of ecosystem services to such transitions.

The CCS of farmland was cross-sensitive to conversion to other land types (Fig. 6b). During the study period, the CCS values of farmland to desert and mining land changed from positive to negative, indicating that the conversion of harvested farmland to the above-mentioned land types had a catalytic to inhibitory effect on the change in ESV. Similarly, the CCS values for farmland versus woodland and watershed changed from negative to positive, indicating that the conversion between farmland and them changed from inhibitory to facilitative for ESV changes. The negative CCS values for farmland versus grassland and wetlands, both of which have an increasing trend, indicate that the conversion between farmland and grassland and wetlands has a less inhibitory effect on ESV change. Similarly, the same is true for the conversion between farmland and construction land, where the CCS value between farmland and construction land changes from 0.96 to 0.22, indicating that the ecosystem becomes progressively less sensitive to such conversion.

The CCS of woodland conversions with other land types (Fig. 6c). During the study period, the CCS values for woodland versus grassland and desert changed from positive to negative, indicating that the conversion between woodland and the land types changed from a facilitating to an inhibiting effect on ESV changes. Similarly, the CCS values for woodland and desert changed from positive to negative, indicating that the conversion between woodland and them changed from a facilitative to an inhibitory effect on ESV change. The negative CCS values for woodland versus construction land, wetlands and waters are in an increasing trend for woodland versus construction land and wetlands, and a decreasing trend for woodland versus waters, indicating that the conversion between woodland and construction land and wetlands has a less inhibitory effect on ESV change, and the conversion between woodland and waters has a more inhibitory effect on ESV change.

The CCS of grassland conversions with other land types (Fig. 6d). The CCS values for transitions between grassland and construction land, waters and mining land changed from negative to positive, indicating that the net change between grassland and the above land types changed from an inhibitory to a facilitative effect on changes in ESV. Conversely, the net change between grassland and wetlands changes from a facilitative to a suppressive effect on ESV. The CCS value for the transition between grassland and desert decreased from 11.46 to 0.43, with the net change between grassland and desert being less sensitive to changes in ESV.

The CCS of construction land conversions with other land types (Fig. 6e). The CCS for conversion between construction land and wetlands was 0, indicating that no conversion occurred between construction land and wetlands. The CCS for the conversion between construction land and waters decreased from 8.43 to 6.19, and the CCS for the conversion between construction land and desert decreased from 1.18 to 0.26 indicating that the net change between construction land and the above land types contributed less to the change in ESV.

The CCS of wetlands conversions with other land types (Fig. 6f). During the study period, the CCS value for conversion between wetlands and deserts was 0, indicating that no conversion of land types occurred between wetlands and deserts. The CCS value for the conversion between wetlands and waters decreased from −0.59 to −3.28, indicating that the net change in wetlands and desert had an increased inhibitory effect on the change in ESV.

The CCS value for the transition between waters and desert changed from 78 to −7.20 (Fig. 6g), indicating that ecosystem services were less sensitive to this transition and that the net change between waters and desert changed from a facilitative to an inhibitory effect on ESV change.

## Discussion

### Impact of land use transition on the spatial distribution of ESV

Correlation analyses have shown that there is a serious imbalance in the spatial distribution of ESV, with the western and eastern parts of the study area and the surrounding towns showing a spatial distribution of High-High Cluster zones, while the central region is dominated by Low-Low Cluster zones. This is also closely related to the local land use situation, with urban areas in the central region being more widely distributed and the main distribution area for mining land^36–38^. This is mainly related to the growth of urbanization in China, as can be seen from the statistical yearbooks and other economic development data for the study area. The study area and surrounding towns have also been affected by rapid urbanization, with the urbanization rates of county-level administrative units included in the study area and surrounding towns being 46.26% and 48.51% respectively as of 2019, an increase of more than 10% over the decade^39–41^. The high urbanization rate is accompanied by a large influx of people into towns and cities, which leads to a surge in demand for urban construction land, resulting in the expansion and even proliferation of construction land, whereas the spatial proximity of farmland to towns and cities makes it inevitable that the expansion of towns and cities will take up some of the farmland, which is the same as the results of previous studies^42–44^.

During the study period, the clustering of ESV in the study area and the surrounding towns changed considerably, with less low–low clustering in the central region and more high–high in the eastern and western regions. This is closely related to changes in land use types, with mining land in the central region declining significantly and the ecological environment moving in a better direction, but still facing great environmental pressure; and the area of forested and watered land in the eastern and western regions increasing, and the environment improving significantly^44–46^.

In the central low–low cluster area, the local government should formulate scientific and reasonable town planning, specify the direction and scope of urban development, and stipulate the land for urban construction and protection, to avoid the situation of disorderly expansion. At the same time, there is a need to improve the land management system, strengthen the management of land-use rights, strictly implement the overall land-use plan and the annual urban land-use plan, and rigorously approve and use land in order to prevent the misuse and indiscriminate use of land. This move will help to protect the ecological environment in the urban periphery and maintain the integrity of the natural ecosystem and biodiversity, thereby avoiding the damage to the ecological environment caused by urban expansion. Concurrently, local governments also need to make efforts to improve rural economic incomes and living standards, and reduce the number of farmers working in cities and the loss of rural labor, to alleviate the pressure brought about by urban expansion. In addition, there is a need to strengthen the planning and management of rare earth mining areas, and to rationally plan the development and use of rare earth mining areas, to reduce the extent of damage to rare earth mining areas. Relevant departments should increase investment in ecological restoration work in rare earth mining areas, including land treatment, vegetation restoration and water restoration, to reduce the adverse impact of mining on the ecological environment. In contrast, in the high and high concentration areas in the eastern and western regions, it is necessary to strengthen the protection of woodland and wetlands, and to improve the forest coverage and vegetation restoration capacity.

### The relationship between land use transition and ESV cross-sensitivity

In terms of the ESV of different ecosystems, woodland and waters were the main contributors to the value of ecosystem services in the whole study area^47–49^. During the study period, the ESV of farmland, grassland, desert, and mining land showed a continuous decreasing trend, while those of woodland and wetlands showed a decreasing and then increasing trend, and those of watershed showed an increasing and then decreasing trend. The ESV changes in the study area were consistent with the trend of land use transformation, with the overall increase and decrease being comparable, and the decrease in ESV was mainly concentrated in the areas with a large increase in mining land and construction land.

In terms of the cross-sensitivity coefficients of land use transformation and ESV, the higher ecological cross-sensitivity of land use change in the study area were the conversion of waters to desert, farmland to waters, mining land to waters, wetlands to mining land, and construction land to waters, respectively, which were closely related to the local land use change^50^. Taking mining land as an example, from 2009 to 2014, the largest area of mining land was converted to farmland in the study area^51–53^. From 2014 to 2019, the largest area of mining land was converted to woodland in the study area. In the early stage of the study, the conversion between mining land and grassland, construction land, wetlands, desert, and waters played a greater inhibitory role in the change of ESV, among which, the conversion of mining land to water played the greatest inhibitory role in the change of its own ESV. In the later part of the study, the net change of mining land to woodland and grassland contributed to the change of ESV.

In summary, woodland and waters have a profound impact on the ESV of a region. It is necessary to strengthen the protection of woodland ecosystems and water ecosystems in order to enhance the ESV. Waste water from rare earth mining usually contains high concentrations of heavy metal ions and acidic substances, while waste slag is rich in high levels of heavy metals and radioactive substances. These waste water and slag pose serious hazards to the surrounding ecological environment. The high precipitation in the study area during the rainy season makes it very easy to wash away the waste water and the slag. Therefore, during the establishment of mining sites in rare earth mining areas, it is important to stay away from water sources and native forests to reduce the harm to the environment.

### Impact of mining and remediation of rare earth mining sites on ESV

The expansion of land for construction and mining, and changes in policies for woodland, wetlands, and waters, among others, are important influences on ecological cross-sensitivity under land use change^53–55^. Rare earth mines can cause damage and destruction of topography, vegetation landscape and land during infrastructure and mining, and land occupation and destruction caused by plant and mine facilities, solid waste dumping, extraction of rare earths and secondary geological hazards can lead to land use type transformation^53^.

Native ecosystems are also destroyed in the process of rare earth mining, leading to the loss of habitat for plants and animals and the reduction of biodiversity. Policy guidance has enabled a significant expansion in the area of woodland, wetlands and waters, and a significant increase in the ESV. Government policies play a crucial role in the rehabilitation of rare earth mines^55^. Drawing on the results of past research on rare earth mining areas^56–58^, local governments should formulate relevant laws and policies to regulate the mining and rehabilitation of rare earth mines. These laws and policies should specify mining standards, environmental protection requirements and rehabilitation responsibilities for mines. Also, regulation should be strengthened and effective regulatory mechanisms should be established to ensure that mining complies with the requirements of the laws and policies. In the process of mining rare earth minerals, local governments should require mining enterprises to conduct environmental impact assessments to evaluate the extent of the environmental impact of mining and to formulate corresponding rehabilitation plans, as well as to provide financial support to assist in the rehabilitation of the mines. This may include providing loans, subsidies, or other forms of economic support to the restoration enterprise to ensure that the restoration work is carried out smoothly, and regularly monitoring the progress of the restoration work to ensure that the restoration plan is effectively implemented. This may include acceptance and evaluation of the rehabilitation work and the imposition of appropriate penalties for substandard rehabilitation work.

### Uncertainty and prospects

As an important assessment indicator of ecosystem service function, the change of ESV value quantity characterizes to some extent the degree of impact of human activities on ecosystem^59^. The cross-sensitivity coefficient between land use change and ESV can quantitatively assess the degree and direction of the impact of land use transition on the ESV with a certain degree of credibility^60–62^.

This study focuses on a typical rare earth mining area in the southern hilly mountains to analyze the impact of land use transition on ESV. Limited by the research data, only three periods of land use data in 2009, 2014 and 2019 were selected for this study; however, land use transition is a long-term change process, and it is necessary to combine more years of land use data for precise analysis^63^. In the future, we can try to expand the study area for systematic analysis, combine ESV with spatial analysis^64,65^, explore the spatial distribution pattern, optimize the national land space, and build an ecological security pattern for sustainable development, in order to provide a more accurate reference for related practices.

## Conclusions

From 2009 to 2019, the land use types in the study area were mainly woodland and farmland, with the highest proportion of woodland, of which the proportion of woodland and farmland accounted for about 80% or more of the total area. During the study period, the most ESV from mining land to construction land and wetlands were shifted in the study area. The ESV transfer process from mining land to woodland to mining land was the highest. The effect of the conversion of mining land to farmland on ESV changed from a facilitative to an inhibitory effect. Conversely, the conversion of mining land to grassland changes the ESV from inhibitory to facilitative. The conversion between mining land and waters and desert has an inhibitory effect on the change of ESV, and the sensitivity of ecosystem services to such conversion gradually decreases. The amount of ESV change in the study area is more consistent with the trend of land use transformation, and the overall increase and decrease are comparable, and the decrease of ESV is mainly concentrated in the areas with a large increase of mining land and construction land. The spatial distribution of ESV in the study area and surrounding towns is dominated by high–high cluster areas and low–low cluster areas, the number of low–high outlier areas and high–low outlier areas is low, and the level of spatial distribution of ESV in the overall study area and surrounding towns is dominated by similar types of cluster areas. The study area is mainly dominated by low–low cluster areas, while the eastern and western parts of the surrounding towns are dominated by high–high cluster areas. The residence of construction and mining land areas in the central region puts the central region in the low-value area, and the neighboring towns have a large area of forested land and a better ecological environment, so there are more high-value areas.

## Data Availability

Land use data for the study area for the three periods 2009–2019 were obtained from the Jiangxi Province Land Use Change Survey database, in which land use data is privately owned and not available. Some statistical yearbook data are available, please contact the first author of the article.
